# Investigation of the cell composition and gene expression in the delayed-type hypersensitivity tuberculin skin test

**DOI:** 10.1186/s40779-023-00450-2

**Published:** 2023-03-16

**Authors:** Hui-Juan Duan, Hong-Qian Chu, Ting-Ming Cao, Guang-Ming Dai, Na Tian, Gang Sheng, Zhao-Gang Sun

**Affiliations:** Beijing Key Laboratory in Drug Resistant Tuberculosis Research, Beijing Tuberculosis and Thoracic Tumor Research Institute, Beijing Chest Hospital, Capital Medical University, Beijing, 101149 China

**Keywords:** Tuberculin skin test, Delayed-type hypersensitivity, Single-cell RNA sequencing

Dear Editor,

The tuberculin skin test (TST) reagents have continuously improved, with the ESAT6-CFP10 (EC) test having recently been introduced, but are seldom based on the direction of the delayed-type hypersensitivity (DTH) mechanism. Previous studies only partially showed the infiltration and activation of immune cells and the production of cytokines of the skin induration [1, 2], and lack the detailed measurements of cell proportions and gene expression in the DTH response. Therefore, in this study, we revealed the comprehensive characteristics of DTH by single-cell RNA sequencing (scRNA-seq) in the guinea pig tuberculosis (TB) model [Experimental Animal Welfare Ethics Committee, Beijing Tuberculosis and Thoracic Tumor Research Institute (2021–064)].

A denser cell infiltrate was stained in the dermis after exposure to EC or purified protein derivative (PPD) than after exposure to phosphate buffered saline (PBS), with the densest infiltrate observed after exposure to PPD. The dermal thickness after PPD exposure [(1768.3 ± 130.9) μm] was significantly higher than that after PBS [(1442.94 ± 77.73) μm] or EC [(1477.52 ± 95.93) μm] exposure (*P* < 0.01, Additional file 1: Fig. S1). Moreover, the area of redness and swelling induced by EC in skin induration was broader than that induced by PPD, consistent with previous studies [3]. These results verified the difference in the DTH responses induced by EC and PPD.

To better understand the DTH induced by EC and PPD, scRNA-seq was performed with skin induration (Additional file 1: Methods). After stringent quality control (Additional file 1: Fig. S2), the transcriptomes of 33,074 cells were obtained and analyzed (Fig. 1a, Additional file 1: Table S1). Then, through SingleR annotation, 10 different major cell types were identified (Fig. 1b, Additional file 1: Fig. S3), among which the controversial cell type of hepatocytes was identified due to the limitation of ImmGen databases being based on mice rather than guinea pigs. Based on established marker genes (Fig. 1c), with a definite correlation between different clusters (Additional file 1: Fig. S4), the cells in the merged dataset were identified as fibroblasts, endothelial cells (Endos), monocytes, T cells, macrophages, natural killer (NK) cells, B cells, granulocytes and dendritic cells (DCs), and the composition of the main cell types, such as T cells, in the EC induration samples differed from that in the PPD induration samples (Fig. 1d, Additional file 1: Fig. S5). Although the reactivity of Endos, NK cells, and B cells in guinea pigs differed slightly from that of the corresponding human cells in the induration samples, most were consistent (Fig. 1b), indicating that the guinea pig is a suitable TST model, with skin architecture, physiology, and immunopathology similar to those in human beings [4].

**Fig. 1:**
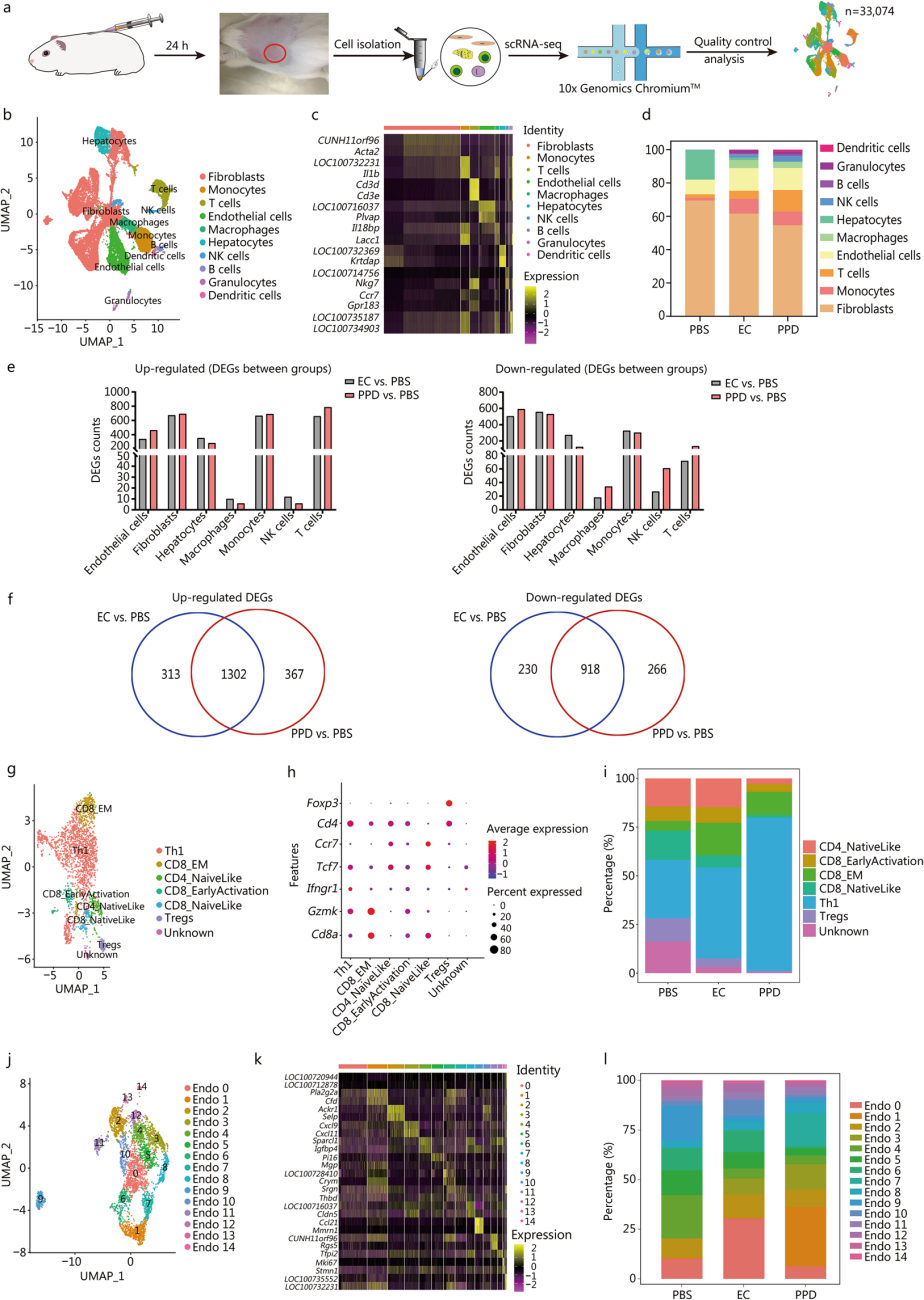
Characteristics of DTH based on the TST.** a** Schematic of the experimental procedure for scRNA-seq. **b** UMAP plot showing ten cell types of indurations. **c** Expression of representative genes for each cell type. **d** Percentage of different cell types of samples with PBS, EC and PPD. **e** Counts of up-regulated and down-regulated DEGs for each cell type in induration. **f** Number of shared up-regulated and down-regulated DEGs between EC and PPD groups. **g** Seven cell subtypes of T cells. **h** Expression of marker genes for T cell subtypes. **i** Percentage of T cell subtypes in PBS, EC and PPD samples. **j** UMAP plot showing 15 cell subtypes in Endos. **k** Expression of representative genes for each subtype in Endos. **l** Percentage of Endos subtypes in PBS, EC and PPD samples. DEGs differentially expressed genes, EC ESAT6-CFP10, PBS phosphate buffered saline, PPD purified protein derivative, Endos endothelial cells, NK cells natural killer cells, UMAP uniform manifold approximation and projection

Moreover, we identified 1615 up-regulated and 1148 down-regulated differentially expressed genes (DEGs) in the EC sample, and 1669 up-regulated and 1184 down-regulated DEGs in the PPD sample (Fig. 1e, Additional file 1: Fig. S6, Additional file 2). Most of the DEGs were common to the EC and PPD samples (Fig. 1f), and 110 up-regulated DEGs were common to at least four identified cell types (Additional file 3). These DEGs were related mainly to the adaptive immune and immune response pathway (Additional file 1: Figs. S7, S8 and S9, Additional file 4), indicating the complex immune interactions during the DTH response between different cell types.

Then, T cells from all samples were clustered unsupervised based on the specific markers as previous reports [5] and 7 cell subpopulations were identified (Additional file 1: Fig. S10): Th1 cells, Tregs, effector-memory CD8^+^ T (CD8_EM) cells, naive CD4^+^ T-like (CD4_NaiveLike) cells, naive CD8^+^ T-like (CD8_NaiveLike) cells, CD8_EarlyActivation cells and “unknown cells” (unable to be characterized based on specific marker genes) (Fig. 1g, h). The ratio of CD4^+^ to CD8^+^ cells was 2 to 1 in the EC sample, but 5 to 1 in the PPD sample (Fig. 1i). The functions of the DEGs were also analyzed (Additional file 1: Fig. S10).

The Endos were significantly increased in the EC and PPD groups (Fig. 1d), and could be categorized into 15 clusters (Fig. 1j–l, Additional file 1: Fig. S11). The functions of the DEGs are analyzed (Additional file 1: Figs. S12 and S13). Moreover, various cytokines were up-regulated in Endos, suggesting that Endos may play more important roles in DTH than previously believed (Additional file 1: Fig. S6).

In conclusion, the heterogeneity of cells and their transcriptomes involved in DTH were first clarified by scRNA-seq in the current study, which determined the immune microenvironment during the DTH response (Additional file 1: Fig. S14). Deciphering the reactivity of different types of cells in DTH could robustly guide the improvement of TST reagents. The most suitable components of the ideal TST reagent are achievable, for example, EC instead of the complex PPD used in TB diagnosis.

## Supplementary Information

**Additional file 1**. Methods.** Fig. S1** Characteristics of skin induration. **Fig. S2** Quality control of all cells acquired by scRNA-seq. **Fig. S3** Cellular diversity and heterogeneity of skin induration. **Fig. S4** Correlation analysis on cell clusters and distribution of cell population. **Fig. S5** Cellular diversity of skin induration. **Fig. S6** DEGs among different cell types caused by EC and PPD. **Fig. S7** Functions of up-regulated DEGs shared by EC and PPD. **Fig. S8** Functions of down-regulated DEGs shared by EC and PPD. **Fig. S9** GO and KEGG enrichment analysis of DEGs in EC and PPD samples. **Fig. S10** Expression patterns and transcriptional regulatory networks of T cells. **Fig. S11** DEGs in Endos in DTH. **Fig. S12** GO and KEGG terms of up-regulated DEGs in Endos. **Fig. S13** GO and KEGG terms of down-regulated DEGs in Endos. **Fig. S14** Scheme of DTH stimulated by different antigens. **Table S1** Results of quality control.

**Additional file 2**. Up-regulated and down-regulated DEGs in total cells in EC or PPD

**Additional file 3**. Up-regulated DEGs shared by EC and PPD expressed in at least four cell types

**Additional file 4**. GO and KEGG terms enriched with up-regulated and down-regulated DEGs in EC or PPD

## Data Availability

The datasets and materials used in this study are available from the corresponding author on reasonable request.

## Abbreviations

DCs: Dendritic cells
DEGs: Differentially expressed genes
DTH: Delayed-type hypersensitivity
EC: ESAT6-CFP10
Endos: Endothelial cells
NK cells: Natural killer cells
PBS: Phosphate buffered saline
PPD: Purified protein derivative
scRNA-seq: Single-cell RNA sequencing
TST: Tuberculin skin test
