# Supplementary material for: Investigation of the cell composition and gene expression in the delayed-type hypersensitivity tuberculin skin test
Source: Mil Med Res. 2023 Mar 16;10:14. doi: 10.1186/s40779-023-00450-2 (PMC10018871; doi:10.1186/s40779-023-00450-2)
Supplement: Supplementary file 1 — Additional file 1. Methods. Fig. S1 Characteristics of skin induration. Fig. S2 Quality control of all cells acquired by scRNA-seq. Fig. S3 Cellular diversity and heterogeneity of skin induration. Fig. S4 Correlation analysis on cell clusters and distribution of cell population. Fig. S5 Cellular diversity of skin induration. Fig. S6 DEGs among different cell types caused by EC and PPD. Fig. S7 Functions of up-regulated DEGs shared by EC and PPD. Fig. S8 Functions of down-regulated DEGs shared by EC and PPD. Fig. S9 GO and KEGG enrichment analysis of DEGs in EC and PPD samples. Fig. S10 Expression patterns and transcriptional regulatory networks of T cells. Fig. S11 DEGs in Endos in DTH. Fig. S12 GO and KEGG terms of up-regulated DEGs in Endos. Fig. S13 GO and KEGG terms of down-regulated DEGs in Endos. Fig. S14 Scheme of DTH stimulated by different antigens. Table S1 Results of quality control. [file 40779_2023_450_MOESM1_ESM.docx]

**Methods**

**Experimental object**

*Mycobacterium tuberculosis* (MTB) standard strain H37Rv (ATCC 27294) was obtained from the clinical data and sample resource database of Beijing Tuberculosis in Beijing Chest Hospital affiliated to Capital Medical University.

EC protein was expressed and purified, and the primer sequences are shown in **Table 1**; SDS-PAGE showed the final purification level, which is presented in **Fig. 1**. TB-PPD (50 IU/ml) was obtained from Beijing Sanroad Biological Products Co., Ltd..

**Table 1** Primers used in this study

| **Gene** | **Forward primer (5’-3’)** | **Reverse primer (5’-3’)** |
| --- | --- | --- |
| *ESAT6-CFP10* (EC) | P1: CCGGATCCATGGCAGAGATGAAGAC  P3: GGTGGCGGTGGAAGCGGCGGTGGCGGAAGCGGCGGTGGCGGCAGCATGACAGAGCAGCAGTGGAATTTCGCGG | P2: GCTGCCGCCACCGCCGCTTCCGCCACCGCCGCTTCCACCGCCACCGAAGCCCATTTGCGAGGACAGCGCCT  P4: CCAAGCTTTGCGAACATCCCAGTGA |
| *Cxcl8* | AGTGCTGTGCGAGGGTATGGTC | GGGTGGAAAGGTGTGGTGTGAATC |
| *Cxcl10* | TTCCAGAACAATACGCTGCACCTG | TGATCTCAACTCGTGGGCAAGATTG |
| *Serping1* | GAGCACACCAGGCACAAGATCAG | GGTAGATGGCGTTGAGCAGCAC |
| *Il18bp* | GAACGCTGACCTTGTCCTGTATGG | GCCTGGGAGGTGCTCAATGAAG |
| *LOC100713317* | TCGTTGCTGCTGTGATTCTCCTTG | AGAGCCCTGGTCACTGTCATCC |
| *Krtdap* | TCCTTCCCGCTGTGACTCTTCTC | GGTAGTTTCTTCCTCACGCTTCTCC |
| *LOC100728410* | ACAGCAAGGACAAGGGCAAATGG | CAGTGGCAATGAGCAGCAGGTC |
| *Lgals* | GGACGGCTTCTCGCTCAATGATG | CAGGGTAGGCACCAGGATAGGC |
| *Ly6d* | CAGTGGAATCTCTGGCAGGAAACC | CAGCTTCTCATTGCACAGGTCCTC |

**Fig. 1** Purified protein of EC. EC ESAT6-CFP10

**Experimental animals and groups**

A total of 9 specific-pathogen-free (SPF) grade female Hartley guinea pigs [Beijing Vital River Laboratory Animal Technology Co., Ltd., SCXK (Beijing) 2016-0011], weighing 250 – 300 g, and divided into three groups: PBS, EC and PPD, with three in each group. The experiment was divided into two parts: 1) For TST. The guinea pig’s back was depilated, and the injection site was wiped with alcohol cotton. PBS, EC protein (diluted with PBS to 5 μg/ml) and TB-PPD (50 IU/ml) were injected intradermally in a 0.1 ml volume. A small colliculus appeared at the injection site. The biological experiment was repeated 3 times. 2) For flow cytometry and single-cell RNA sequencing (scRNA-seq). The PBS, EC and PPD were re-injected into each group after the induration disappeared (about 1 week). The guinea pigs were killed and the indurations were excised after 24 h for subsequent experiments.

**Establishment of the TB infection model**

The MTB standard strain H37Rv (ATCC 27294) was cultured on neutral modified Lowenstein-Jensen medium at 37 ℃ for 3 – 4 weeks. After measuring the optical density (OD), 0.5 ml (2×10^7^ CFU/ml) was injected subcutaneously into the inner thigh/inguinal area of the guinea pigs for 4 weeks.

**TST**

The diameter (the average of length plus width) of each skin induration was measured 24 h and 48 h after the injection.

**Hematoxylin-eosin (HE) staining**

Skin tissues isolated from guinea pig dorsal skin were fixed with 4% paraformaldehyde. HE staining was performed using the paraffin section method. The slices obtained were processed by Wuhan Servicebio Biotechnology Co., Ltd.. The data were analyzed with CaseViewer software (version 2.4, 3DHISTECH Ltd., Budapest, Hungary).

**Sample preparation and tissue dissociation**

After 24 h, the guinea pigs were killed, and the induration was excised. All biopsy skin samples were immediately processed for flow cytometry and scRNA-seq analysis (Berry Genomics, Beijing, China). The skin tissues were transferred to a 50-ml centrifuge tube and washed with precooled PBS at 4 °C. The tissues were placed in a precooled dish on ice, cut into 2 to 4-mm pieces, and then all transferred to an Eppendorf tube containing an enzyme mix [Collagenase I (1 mg/ml) + Collagenase IV (0.5 mg/ml) + Collagenase V (0.5 mg/ml) (Solarbio) + Collagenase-neutral protease (Roche) + DNase *I* (Solarbio)]. A gentleMACS^TM^ Octo Dissociates with Heaters was used for 4 h for dissociation and digestion. After dissociation, the cells were filtered through a 70-μm filter membrane. The cell suspension was centrifuged at 300×*g* for 7 min at 4 °C, and the supernatant was discarded. Erythrocytes were removed using precooled BD Pharm Lyse^TM^ lysing buffer at 4 °C, and then, the sample was washed twice with precooled PBS + 0.04% bovine serum albumin (BSA). Finally, dead cells were removed with a dead cell removal kit (Miltenyi Biotec).

**Flow cytometry**

Fixability Stain 660 (BD) was used to label dead cells. Following incubation for 10 min, the cells were centrifuged at 300×*g* for 5 min at 4 °C. Then, the dissociated skin cells were labeled with antibody (FITC-conjugated mouse anti-guinea pig CD45, MA528382, Invitrogen) in PBS for 25 min at 4 °C. After incubation, the cells were centrifuged at 300×*g* for 5 min at 4 °C, washed twice with PBS, and resuspended. The cells were assessed on an LSRFortessa flow cytometer (BD Biosciences), and the data were analyzed with FlowJo software.

**Single-cell cDNA and library preparation**

Single-cell cDNA, library preparation, and 3’-end scRNA-seq were performed by Berry Genomics (Beijing, China). Cell labeling was performed based on the 10× Genomics Chromium^TM^ system. Single-cell RNA-seq libraries were prepared using Chromium Single Cell 3ʹ Reagent Kit v3 (10× Genomics) according to the manufacturer’s instructions. The single-cell suspension was washed with 0.04% BSA DPBS three times. The cell number and concentration were confirmed with a Countess^®^ Ⅱ Automated Cell Counter. Cell labeling was performed based on the 10× Genomics Chromium^TM^ system. Gel beads with barcode information were mixed with cells and enzymes, and separated oil beads were to form gel beans-in-emulsion (GEMs). In each GEM, the gel beads were dissolved, primers were released, and any composition cells are lysed. Primers that contained an Illumina TruSeq Read 1 (read 1 sequencing primer), a 16-nt 10× barcode, a 12-nt unique molecular identifier (UMI) and 30-nt poly(dT) sequence were mixed with the cell lysate and Master Mix containing reverse transcription RT reagents. Incubation of the GEMs produced branched, full-length cDNA from polyadenylated mRNA. Then, the cDNA was collected and amplified, and the sequencing library was constructed according to the standard process through Illumina sequencing library construction. The libraries were sequenced on a NovaSeq platform (Illumina) to generate 150-bp paired-end reads according to the manufacturer’s instructions.

**Quality control**

Raw data (raw reads) of fastq files were assembled from the raw BCL files using the Illumina bcl2fastq converter. Raw data were first processed through primary quality control. The monitored quality assessment parameters were as follows: 1) an N value greater than 3; 2) a proportion of bases with quality values less than 5 totaling more than 20%; and 3) adapter sequences. All downstream analyses were based on clean high-quality data.

**Generation and analysis of single-cell transcriptomes**

Raw reads were demultiplexed and mapped to the reference genome through the 10× Genomics Cell Ranger pipeline (https://support.10xgenomics.com/single-cell-geneexpression/software/pipelines/latest/what-is-cell-ranger) using default parameters. All downstream single-cell analyses were performed using Cell Ranger and Seurat [1] unless stated otherwise. In brief, for each gene and each cell barcode (filtered by Cellranger), UMIs were counted to construct digital expression matrices. Through secondary filtration by Seurat, a gene expressed in more than 3 cells was considered to be expressed, and each cell was required to express at least 200 genes meeting this criterion. Some of the foreign cells were eliminated. Taking with fastq files, Cellranger count performs alignment, filtering, barcode counting, and UMI counting. The package uses the Chromium cell barcodes to generate feature barcode matrices with Cellranger count or Cellranger aggr and reruns the dimensionality reduction, clustering, and gene expression algorithms on the basis of Cellranger default parameter settings. For secondary analysis of gene expression, we used Seurat. The Seurat package normalizes data, performs dimensionality reduction, clustering, and identifies differential expression. We used the Seurat alignment method called canonical correlation analysis (CCA) [2] to perform an integrated analysis of the datasets. For clustering, highly variable genes were selected, and the principal components were identified based on the genes used to build a graph, which was segmented at a resolution of 0.6. The correlation between clusters was analyzed by calculating the Pearson correlation coefficient between clusters.

**Gene Ontology (GO) and Kyoto Encyclopedia of Genes and Genomes (KEGG) pathway enrichment analyses**

GO and KEGG enrichment analyses of DEG sets were performed with the GO seq R and KOBAS 3.0 packages, respectively. GO terms with adjusted *P*-values below 0.05 were considered to be significantly enriched with DEGs.

**Quantitative real-time PCR (qRT-PCR)**

Total RNA was isolated with TRIzol reagent (TransGen Biotech). First-strand cDNA was synthesized using a FastKing RT Kit (with gDNase) (Tiangen Biotech). Gene-specific primer pairs were designed with Primer Premier 5.0 software (**Table 1**). **qRT-PCR** was performed using PowerUp^TM^ SYBR^TM^ Green Master Mix (Applied Biosystems) according to the manufacturer’s protocol.

**Statistical analysis**

Statistical analyses were performed using GraphPad Prism software (version 8.0.1, GraphPad Software, San Diego, CA) and SPSS software (version 22.0, IBM Corp, Armonk, NY, US). The data represent the *M* (*Q*_1_, *Q*_3_) or mean ± standard deviation (SD). Wilcoxon rank sum test, one-way analysis of variance (ANOVA) and a two-tailed, unpaired *t*-test were performed to compare the values between different groups based on the quantitative data. *P*-values < 0.05 was considered statistically significant.

**References**

[1] Macosko EZ, Basu A, Satija R, Nemesh J, Shekhar K, Goldman M, et al. Highly parallel genome-wide expression profiling of individual cells using nanoliter droplets. Cell. 2015,161(5):1202-14.

[2] Butler A, Hoffman P, Smibert P, Papalexi E, Satija R. Integrating single-cell transcriptomic data across different conditions, technologies, and species. Nat Biotechnol. 2018,36(5):411-20.

**Fig. S1** Characteristics of skin induration. **a** Hematoxylin-eosin (HE) staining analysis of histological features to the PBS, EC and PPD in skin indurations. Scale bars, 500 μm. Dermis thickness of induration is presented as the mean ± standard deviation (SD) (*n* = 5). One-way analysis of variance (ANOVA). **b** The size of skin induration to the PBS, the protein of EC and PPD in guinea pig’s back. Data are presented as the *M* (*Q_1_, Q_3_*) (*n* = 9). Wilcoxon rank sum test. **c** Flow cytometry revealed the proportion of CD45^+^-labeled leukocytes of the protein of EC and PPD in skin indurations. ^**^*P* < 0.01, ^***^*P* < 0.001, ns not significant; PBS phosphate buffered saline, EC ESAT6-CFP10, PPD purified protein derivative

**Fig. S2** Quality control of all cells acquired by scRNA-seq. **a** Visualize the distribution map of base sequencing base content (left) and quality value (right) according to fastqc, including samples of PBS, EC and PPD. The distribution map of base content showed sequence of bases in reads pair in abscissa and the proportion of different bases in ordinate, where N is the unknown base. The distribution map of quality value showed sequence of bases in reads pair in abscissa and the base mass in ordinate. The color depth represents the proportion of bases at this position in different base quality gradients in all reads. The higher the proportion of high-quality bases, the better the sequencing data. **b** Multiple cell identification plot, left is the multicellular score histogram of the observed transcriptomes; right is the multicellular score histogram of the simulated doublets, including samples of PBS, EC and PPD. **c** Low-quality cell filtration image including samples of PBS, EC and PPD. The left image is before quality control, and the right image is after quality control. Each point represents a cell, the ordinate of nFeature_RNA represents the number of genes expressed in each cell, the ordinate of nCount_RNA represents the total number of UMIs in each cell and the ordinate of percent.mt represents that the UMI of mitochondrial genes in each cell accounts for the total number of UMIs in the cell. The ratio of UMI, the abscissa indicates the abundance of cells at each ordinate value. PBS phosphate buffered saline, EC ESAT6-CFP10, PPD purified protein derivative, UMI unique molecular identifier

**Fig. S3** Cellular diversity and heterogeneity of skin induration. **a** UMAP plot showing 38 clusters of skin induration. Cell types are distinguished by different colors. **b** UMAP plot of all cells acquired by scRNA-seq of induration samples in guinea pigs skin stimulating by PBS, EC and PPD. Cells obtained from different samples were labeled in different colors. **c** Heatmap showing the expression of representative genes for each cluster. Clusters are distinguished by different colors. **d** UMAP plots showing the representative genes of each cluster in guinea pig’s skin induration from different samples, including PBS, EC and PPD. The color key from gray to purple indicates low to high gene expression levels. scRNA-seq single-cell RNA sequencing, PBS phosphate buffered saline, EC ESAT6-CFP10, PPD purified protein derivative, UMAP uniform manifold approximation and projection

**Fig. S4** Correlation analysis on cell clusters and distribution of cell population. **a** Pearson correlation coefficient analysis showed the correlation between different clusters (clusters 0 – 37). **b** Single R-heatmap showed the classification of cell populations. Each column is a cluster and a total of 38; each row is a cell type which database has a total of 18 types. During annotation, each cluster is scored. Each cluster has a score corresponding to each cell type, which is the color block in the middle of the heatmap. The score corresponds to the right color bar, yellow has the highest score and dark blue has the lowest score. NK cells natural killer cells

**Fig. S5** Cellular diversity of skin induration. **a** UMAP plots showing cell types of guinea pig skin stimulated by different antigens, including PBS, EC and PPD. Cell types are distinguished by different colors. **b** Dot plot showing the expression of representative genes for each cell type with different samples, including PBS, EC and PPD. **c** The histogram shows the number of different cell types in the three samples. Different samples were labeled in different colors. **d** UMAP plots showing the representative genes of each cell type in guinea pigs skin from different sample, including PBS, EC and PPD. The color key from gray to purple indicates low to high gene expression levels. PBS phosphate buffered saline, EC ESAT6-CFP10, PPD purified protein derivative, UMAP uniform manifold approximation and projection, NK cells natural killer cells

**Fig. S6** DEGs among different cell types caused by EC and PPD. **a** Histogram showing the number of DEGs in different cell types after cluster analysis, including PBS, EC and PPD. Cell types are distinguished by different colors. **b** Histogram showing the counts of up-regulated and down-regulated DEGs for each cell type in guinea pig skin with EC vs. PBS (left) and PPD vs. PBS (right). **c** Volcano plot showed the DEGs of different cell types in EC vs. PBS and in PPD vs. PBS. The genes marked on the graph are those with |log_2_ FC| > 1, *P*_adj < 0.05. The up-regulated DEGs were noted in red and down-regulated DEGs were noted in blue. **d** Up-regulated genes shared by EC and PPD (respectively top 20 genes) expressed in at least four cell types, related to **Additional file 3**. **e** Expression of some up-regulated and down-regulated genes in immune cells verified by qRT-PCR. Data are presented as mean ± SD (*n*=3). Two-sample unpaired *t*-test. ^**^*P* < 0.01, ^***^*P* < 0.001, ns not significant; DEGs differentially expressed genes, PBS phosphate buffered saline, EC ESAT6-CFP10, PPD purified protein derivative, qRT-PCR quantitative real-time PCR, NK cells natural killer cells

**Fig. S7** Functions of up-regulated DEGs shared by EC and PPD. Representative GO and KEGG terms of up-regulated DEGs shared by EC and PPD after compared with PBS, respectively. DEGs differentially expressed genes, EC ESAT6-CFP10, PPD purified protein derivative, GO Gene Ontology, KEGG Kyoto Encyclopedia of Genes and Genomes, TNF tumor necrosis factor, NOD nucleotide oligomerization domain, BP Biological Process, CC Cellular Component, MF Molecular Function

**Fig. S8** Functions of down-regulated DEGs shared by EC and PPD. Representative GO and KEGG terms of down-regulated DEGs shared by EC and PPD after compared with PBS, respectively. DEGs differentially expressed genes, EC ESAT6-CFP10, PPD purified protein derivative, GO Gene Ontology, KEGG Kyoto Encyclopedia of Genes and Genomes, ERK extracellular regulated protein kinases, SMAD drosophila mothers against decapentaplegic, COVID-19 coronavirus disease 2019, ECM extracellular matrix, BP Biological Process, CC Cellular Component, MF Molecular Function

**Fig. S9** GO and KEGG enrichment analysis of DEGs in EC and PPD samples. **a-b.** The circle graphs showed the GO enrichment (left) and KEGG enrichment (right) pathways regulated by up-regulated (**a**) and down-regulated (**b**) DEGs only in EC sample. **c-d**. The circle graphs showed the GO enrichment (left) and KEGG enrichment (right) pathways regulated by up-regulated (**c**) and down-regulated (**d**) DEGs only in PPD sample. Related to **Additional file 4**. DEGs differentially expressed genes, EC ESAT6-CFP10, GO Gene Ontology, KEGG Kyoto Encyclopedia of Genes and Genomes, BP Biological Process, CC Cellular Component, MF Molecular Function

**Fig.** **S10** Expression patterns and transcriptional regulatory networks of T cells. **a** UMAP plot showing 13 clusters of skin indurations in T cells. Cell types are distinguished by different colors. **b** UMAP plot of T cells acquired by scRNA-seq of induration samples in guinea pigs skin stimulating by PBS, EC and PPD. Cells obtained from different samples were labeled in different colors. **c** Dot plot showing the expression of representative genes for each cell type. **d** Counts of cell subtypes of T cells skin induration of PBS, EC and PPD. **e** Volcano plot of DEGs that up-regulated (red) or down-regulated (blue) of T cells after EC (left) and PPD (right) compared with PBS, respectively. The genes marked on the graph are those with |log_2_ FC| > 1, *P*_adj < 0.05. **f** Representative GO terms of up-regulated and down-regulated DEGs in EC vs. PBS and PPD vs. PBS in T cells. **g** Venn plot showing the number of shared up-regulated and down-regulated DEGs between EC vs. PBS (blue) and PPD vs. PBS (red) in T cells. **h** Representative KEGG terms of up-regulated DEGs between EC and PPD, only in EC vs. PBS and only in PPD vs. PBS in T cells. The color keys from red to green, indicate the range of –log_10_ *P*-value. The size of the dots represents the count of genes. DEGs differentially expressed genes, EC ESAT6-CFP10, PBS phosphate buffered saline, PPD purified protein derivative, GO Gene Ontology, KEGG Kyoto Encyclopedia of Genes and Genomes

**Fig. S11** DEGs in Endos in DTH. **a** UMAP plot of Endos acquired by scRNA-seq of induration samples in guinea pigs skin stimulating by PBS, EC and PPD. Cells obtained from different samples were labeled in different colors. **b** The histogram shows the number of different clusters in the three samples (PBS, EC and PPD). Different samples were labeled in different colors. **c** Violin plots illustrating the expression of some marker genes in each cell type. **d** Venn plot showing the number of shared up-regulated (upper) and down-regulated (lower) DEGs between EC vs. PBS (blue) and PPD vs. PBS (red) in Endos. **e-f** The DEGs in EC vs. PBS (**e**) and in PPD vs. PBS (**f**) showed by the heatmap (left) and the volcano plot (right). The color keys from red to blue, indicate the level of gene expression in the heatmap. The up-regulated DEGs were noted in red and down-regulated DEGs were noted in blue in the volcano plot. DTH delayed-type hypersensitivity, DEGs differentially expressed genes, EC ESAT6-CFP10, PBS phosphate buffered saline, PPD purified protein derivative, GO Gene Ontology, KEGG Kyoto Encyclopedia of Genes and Genomes, Endos endothelial cells, scRNA-seq single-cell RNA sequencing

**Fig. S12** GO and KEGG terms of up-regulated DEGs in Endos. Representative GO (**a**) and KEGG (**b**) terms of up-regulated DEGs in both EC and PPD, only in EC vs. PBS and only in PPD vs. PBS in Endos. DEGs differentially expressed genes, EC ESAT6-CFP10, Endos endothelial cells, PBS phosphate buffered saline, PPD purified protein derivative, GO Gene Ontology, KEGG Kyoto Encyclopedia of Genes and Genomes, BP Biological Process, CC Cellular Component, MF Molecular Function

**Fig. S13** GO and KEGG terms of down-regulated DEGs in Endos. Representative GO (**a**) and KEGG (**b**) terms of down-regulated DEGs in both EC and PPD, only in EC vs. PBS and only in PPD vs. PBS in Endos. DEGs differentially expressed genes, EC ESAT6-CFP10, Endos endothelial cells, PBS phosphate buffered saline, PPD purified protein derivative, GO Gene Ontology, KEGG Kyoto Encyclopedia of Genes and Genomes, BP Biological Process, CC Cellular Component, MF Molecular Function

**Fig. S14** Scheme of DTH stimulated by different antigens: PBS, EC and PPD. The skin consists of the epidermis and dermis. The epidermis is divided into the basal layer, spinous layer, granular layer and cuticle. After stimulation by EC and PPD, the proportion of the cell population in skin tissue changed to a certain extent. T cells in the figure include other subsets other than CD4^+^ Th1 cells. EC ESAT6-CFP10, Endos endothelial cells, PBS phosphate buffered saline, PPD purified protein derivative, DTH delayed-type hypersensitivity, IFN interferon, TNF tumor necrosis factor, LT lymphotoxin, IL interleukin

**Table S1** Results of quality control

| Analysis by Cell Ranger |  | PBS | EC | PPD |
| --- | --- | --- | --- | --- |
| **The quality of sequencing data** |  |  |  |  |
| Valid barcides |  | 97.6% | 97.5% | 97.4% |
| Sequencing saturation |  | 74.4% | 60.8% | 58.6% |
| Number of reads |  | 709,022,192 | 880,053,942 | 833,063,170 |
| Q30 bases in barcode |  | 95.0% | 96.2% | 95.9% |
| Q30 bases in RNA read |  | 91.2% | 92.6% | 92.0% |
| Q30 bases in UMI |  | 94.1% | 96.0% | 95.6% |
| **Contrast ratio** |  |  |  |  |
| Reads mapped confidently to genome |  | 86.7% | 86.7% | 86.8% |
| Reads mapped confidently to transcriptome |  | 52.2% | 53.2% | 53.0% |
| Reads mapped antisense to gene |  | 0.7% | 0.9% | 1.0% |
| **Cell number and median of gene** |  |  |  |  |
| Estimated number of cells |  | 7732 | 13,446 | 12,742 |
| Mean reads per cell |  | 91,700 | 65,451 | 65,379 |
| Median genes per cell |  | 2148 | 1683 | 1983 |
| Fraction reads in cells |  | 90.1% | 82.7% | 84.9% |
| Total genes detected |  | 16,944 | 16,994 | 16,891 |
| Median UMI counts per cell |  | 8112 | 6594 | 7368 |
| **Number of cells before and after cell filtration** |  |  |  |  |
| All cell |  | 7732 | 13,446 | 12,742 |
| Multiplets |  | 122 | 49 | 98 |
| MT cell |  | 180 | 182 | 206 |
| nFeature |  | 61 | 1 | 8 |
| nCount |  | 0 | 0 | 0 |
| Keep cell |  | 7421 | 13,215 | 12,438 |
| Keep genes |  | 16,021 | 16,140 | 15,916 |

*PBS* phosphate buffered saline, *EC* ESAT6-CFP10, *PPD* purified protein derivative, *UMI* unique molecular identifier
